# Landscape-scale spatial heterogeneity in phytodetrital cover and megafauna biomass in the abyss links to modest topographic variation

**DOI:** 10.1038/srep34080

**Published:** 2016-09-29

**Authors:** Kirsty J. Morris, Brian J. Bett, Jennifer M. Durden, Noelie M. A. Benoist, Veerle A. I. Huvenne, Daniel O. B. Jones, Katleen Robert, Matteo C. Ichino, George A. Wolff, Henry A. Ruhl

**Affiliations:** 1 National Oceanography Centre, University of Southampton Waterfront Campus, European Way, Southampton SO14 3ZH, UK; 2 Ocean and Earth Science, University of Southampton, National Oceanography Centre Southampton, European Way, Southampton SO14 3ZH, UK; 3 School of Environmental Sciences, University of Liverpool L69 3BX, UK

## Abstract

Sinking particulate organic matter (POM, phytodetritus) is the principal limiting resource for deep-sea life. However, little is known about spatial variation in POM supply to the abyssal seafloor, which is frequently assumed to be homogenous. In reality, the abyss has a highly complex landscape with millions of hills and mountains. Here, we show a significant increase in seabed POM % cover (by ~1.05 times), and a large significant increase in megafauna biomass (by ~2.5 times), on abyssal hill terrain in comparison to the surrounding plain. These differences are substantially greater than predicted by current models linking water depth to POM supply or benthic biomass. Our observed variations in POM % cover (phytodetritus), megafauna biomass, sediment total organic carbon and total nitrogen, sedimentology, and benthic boundary layer turbidity, all appear to be consistent with topographically enhanced current speeds driving these enhancements. The effects are detectable with bathymetric elevations of only 10 s of metres above the surrounding plain. These results imply considerable unquantified heterogeneity in global ecology.

The ocean basin-scale distribution and abundance of life on the abyssal seafloor is set extensively by the supply of sinking particulate organic matter (POM) from overlying surface waters, a limiting food resource for most deep-sea life^1^,^2^,^3^,^4^. At regional to local scales, it is suggested that bathymetry, habitat terrain type, and lateral transport of POM play important roles in determining the distribution of biomass and biological assemblage composition^5^,^6^,^7^,^8^,^9^. About 85% of the world’s seafloor lies at abyssal depths, and contains many millions of hill features^10^,^11^. If these bathymetric features, rising only 10 s to 100 s of metres above the abyssal plain, support differing quantities of biomass, this intermediate heterogeneity would be important to global biogeochemistry, ecology, and biogeography.

As a result of remineralisation in the water column, particulate organic carbon (POC) flux decreases with increasing water depth^12^. The Martin curve^13^ allows for the estimation of variation in POC flux with water depth (*Z*), i.e. flux ∝ *Z*^−0.7^ (*Z* in metres)^14^. Similarly, the corresponding decline in the expected standing stock of seafloor biomass supported by that flux can be linked to water depth via other studies, e.g. megafauna biomass ∝ 10^−0.4*Z*^ (*Z* in kilometres; Supplementary Text)^3^. The vertical fluxes of POC, particle volume, and particle mass over the Porcupine Abyssal Plain (PAP) are all highly correlated^3^,^15^. These relationships provide a null framework against which to judge the potential impact of abyssal hill terrain on the supply of POM to the seafloor and consequently biomass. In this contribution we assess POM supply as the areal cover (%) of the seafloor by POM aggregates, frequently referred to as phytodetritus, which can be visually distinguished from the seafloor sediment surface^16^,^17^.

Previous studies of PAP abyssal hill sediments have detected reduced organic-carbon and nitrogen content, reduced degradation of proteinaceous organic matter, and a reduced silt and clay fraction (particles <63 μm), relative to surrounding abyssal plain sediments^18^,^19^. Biological observations on abyssal hills of modest elevation (100 to 500 m) have recorded a 3-fold increase in megafauna biomass relative to the adjacent abyssal plain^18^. Both latter sets of observations are consistent with increased bottom water flows around and over the elevated terrain controlling the parameters measured. In contrast, no difference was detected in the density of fishes between PAP plain and hill terrain^20^. Those studies, and the present contribution, concern environments >3 km below the maximum mixed layer depth of the surface ocean^21^, indicating that there is no plausible mechanism of interaction between local seafloor terrain and overlying primary production.

Here, we test three hypotheses concerning the notion that significant local-scale ecological variations may arise from subtle changes in abyssal topography (i.e. small hills), specifically: (1) that local terrain characteristics are linked to seafloor POM cover; (2) that consequently, invertebrate megafauna biomass will vary in a similar manner; and (3) that sediment organic carbon and silt-clay content may vary in an opposing manner as a result of sediment winnowing over elevated terrain. We reference our observations to a null expectation of a remineralisation with depth-only driven change in organic matter supply and megafauna biomass. Using an exceptionally large photographic dataset, we test these concepts on modest terrain elevations (abyssal hills) on the PAP (4850 m water depth, NE Atlantic 49° 00′ N 16° 30′ W)^22^, collected by an Autonomous Underwater Vehicle (AUV). This study is the first, to our knowledge, to simultaneously quantify POM cover (~92,000 images; 15 ha total) and megafauna biomass (~65,000 images; 9 ha total) over such a large area of deep seafloor.

## Results

### POM Distribution

**Figure 1:**
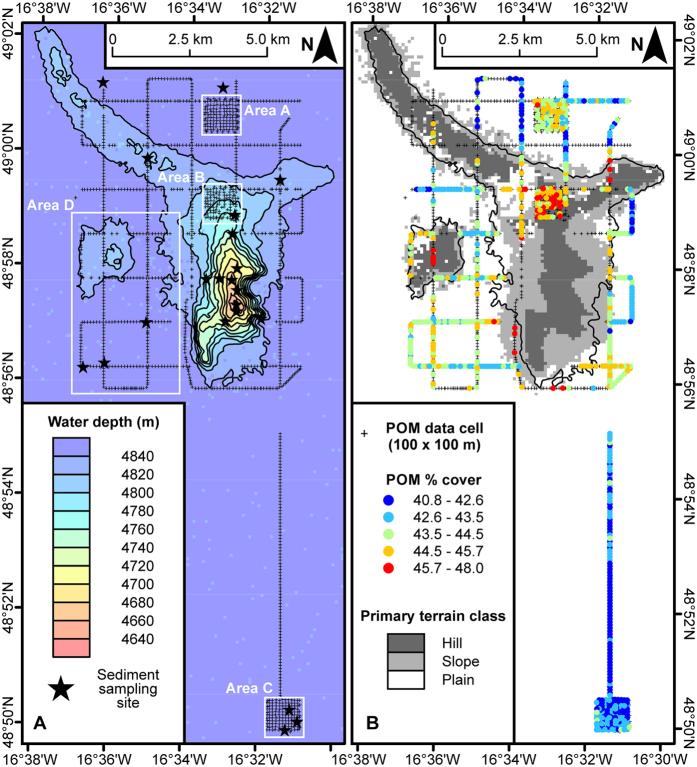
Porcupine Abyssal Plain study area. (**A**) Bathymetry, illustrated as 20 m contours and corresponding colour fill. Track of autonomous vehicle survey indicated (+) as those map cells (100 × 100 m) for which particulate organic matter (POM) data were generated. Sub-areas of particular interest are delimited (Area A, Northern Plain; Area B, Main Hill; Area C, Southern Plain; Area D, small hill and its surrounding plain). Seabed sampling sites are also indicated (*). (**B**) Spatial variation in seabed POM cover is illustrated by colour-coded symbols for those map cells having 50 or more data values. The primary hill-bounding bathymetric contour (4840 m), and the defined primary terrain types are also illustrated. Map projection is UTM WGS 1984 zone 28N (ArcGIS v10.2, Environmental Systems Research Institute); bathymetric contours and primary terrain classes have been edited to remove small features (noise) over the abyssal plain (note, un-edited data were used in all analyses, and are illustrated in Supplementary Fig. 1).

**Figure 2:**
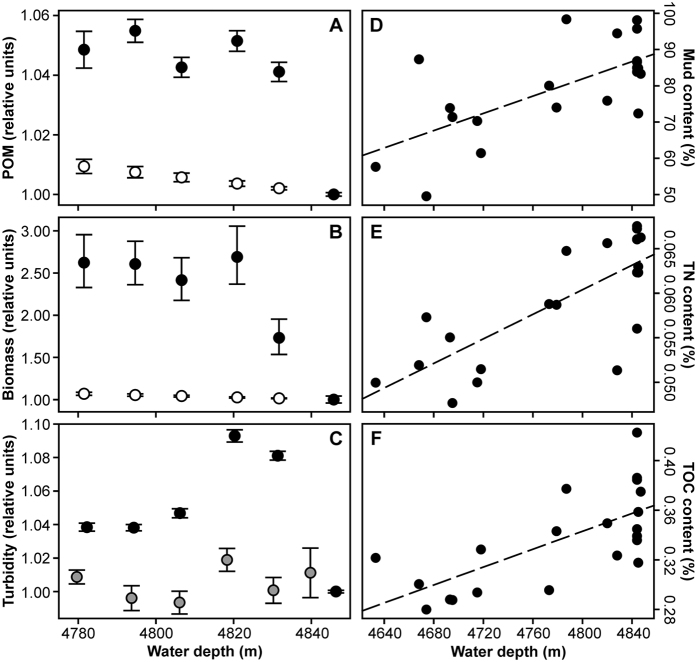
Terrain elevation related variations in megafauna biomass and organic matter supply terms. (**A**) Particulate organic matter (POM) seabed cover (observed mean • and 95% confidence interval; null prediction ○ and 95% CI). (**B**) Megafauna biomass (observed mean • and 95% CI; null prediction ○ and 95% CI). **C.** Turbidity in water column (altitude >10 m, ■), and benthic boundary layer (altitude <10 m, •), as mean and 95% CI. (**A–C**) Values plotted normalised to the abyssal plain (plain value = 1.00). (**D**) Sediment mud content (particles <63 μm, 0–10 mm sediment horizon). (**E**) Sediment total nitrogen content (TN, 0–10 mm horizon). (**F**) Sediment total organic carbon content (TOC, 0–10 mm horizon). (**D–F**) Dashed line represents simple least squares linear fit, all relationships are significant (Spearman’s rank correlation p < 0.02).

**Figure 3:**
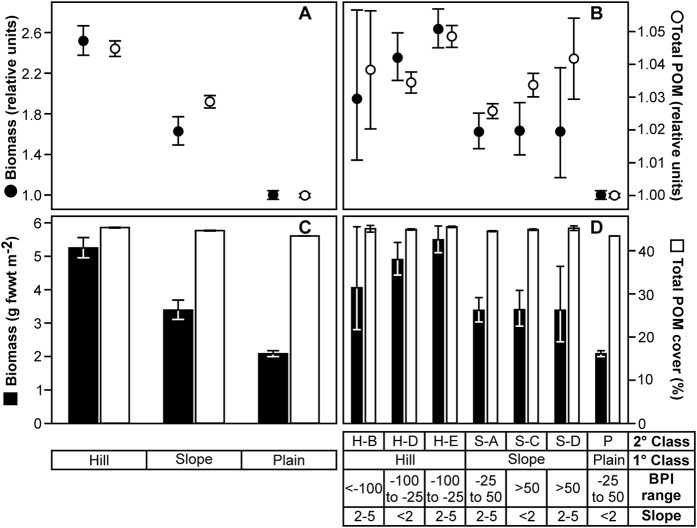
Variation in seafloor total particulate organic matter (POM) cover, and megafauna biomass in primary and secondary terrain classes. Data are illustrated as mean values with 95% confidence intervals: (**A,B**) as normalised to the abyssal plain (plain value = 1.00), and (**C,D**) in natural units. Shown with corresponding tabulation of terrain classification variables, bathymetric position index (BPI) and angle of seabed inclination (Slope, degrees), for primary (1°) and secondary (2°) levels of classification. (H, Hill; S, Slope; P, Plain). (The full classification scheme is presented in Supplementary Fig. 1).

POM cover (Fig. 1B) exhibited highly significant variation between depth bands, with the deepest band (representing the abyssal plain) significantly different from all shallower bands (Table 1, Supplementary Table 1), and the shallower depth bands (hill) having total POM cover 1.04–1.05 times higher than the plain (Fig. 2A). Although the difference was small, the Cohen’s effect size value (*d* = 0.5–0.6) was of “moderate” practical significance. Similarly, a separate assessment of the small hill and surrounding plain in Area D (Fig. 1A) indicated highly significant variation with depth, the deepest band (plain) being significantly different from all shallower bands (Supplementary Table 1). Comparison of the high resolution survey areas (Fig. 1A, Areas A–C) again indicated a highly significant difference between hill and plain areas. A significant difference was also detected between the two plain areas (Areas A and C; Supplementary Table 1). POM cover increased by a factor of 1.03 from the Northern Plain to the Hill (*d* = 0.4, “small-medium” effect), and by a factor of 1.07 from the Southern Plain to the Hill (*d* = 0.8, “large” effect). As detailed in the Methods section, local terrain type was classified by joint consideration of relative local elevation and local seabed slope angle to derive primary and secondary terrain types. The defined primary terrain classes (‘Hill’, ‘Slope’, ‘Plain’) exhibited highly significant differences in POM cover, with significant differences in all pairwise comparisons, Hill the highest cover and Plain the lowest (Table 1, Fig. 3A,C). Similarly, POM cover varied with the defined secondary terrain classes, with the Plain class significantly different from all others (Table 1, Fig. 3B,D).

### Biomass Distribution

Megafauna biomass exhibited highly significant variation between depth bands, with the two deepest bands significantly different from all shallower bands, and from each other (Table 1, Supplementary Table 1). These latter contrasts were of substantial magnitude (*d* = 1.4–1.9, “very large” practical significance). There was a factor of 2.5 increase in megafauna biomass from the plain to the shallowest depth band (Fig. 2B). Similarly, a separate assessment of the small hill and surrounding plain in Area D (Fig. 1A) indicated significant variation with depth, with the top of the small hill having significantly higher biomass than deeper bands (Supplementary Table 1). Comparison of the high resolution survey areas (Fig. 1A, Areas A–C) indicated highly significant variation between hill and plain areas, but no significant difference between the two plain areas (Supplementary Table 1). Primary terrain classes exhibited highly significant differences in biomass, with all three classes significantly different from one another, Hill the highest and Plain the lowest (Table 1, Fig. 3A,C). Similarly, biomass varied among secondary terrain classes, with the ‘Plain’ class significantly different from all others with the exception of the ‘Slope D’ class (Table 1, Fig. 3B,D).

### Turbidity and Seabed Sediments

Benthic boundary layer (BBL, water column <10 m above seafloor) turbidity (suspended particulate load) exhibited a modest but marked increase over the elevated terrain, with values some 1.04–1.09 times higher than the plain (Fig. 2C). Water column turbidity >10 m above the seafloor was broadly consistent over the depth range examined and very similar to BBL turbidity over the abyssal plain (Fig. 2C). Across the 21 sites sampled with a Megacorer (Fig. 1A), sediment mud content (Mud %), total nitrogen content (TN %), and total organic carbon content (TOC %) exhibited significant positive correlations with water depth (Table 2; Fig. 2D–F). Similarly, TN % and TOC % exhibited significant positive correlations with Mud % (p < 0.05, p < 0.01, respectively). Comparisons of all three parameters between abyssal plain (sites >4840 m) and elevated terrain (sites <4840 m) locations indicated significant differences (p < 0.02) in all cases (Table 2). In contrast, the TOC-to-TN ratio (C/N) exhibited no significant correlation with water depth, and no significant difference between abyssal plain and elevated terrain locations (Table 2).

### Environment-POM-Biomass

Links between POM cover, biomass, and water depth were assessed by reference to predictions based on the influence of water depth alone (Fig. 2A,B). In each case, the observed increase in POM cover and biomass at shallower depths was greater than the null framework predicted, with POM cover increasing by a factor of 1.05 and biomass by a factor of 2.5 in comparison to the predicted values of 1.01 and 1.07 respectively. Inter-relationships between POM cover, biomass, and environmental variables were assessed by Spearman’s rank correlation (Table 3). POM cover and biomass exhibited significant correlations with each other and with most other variables tested (Table 3). We also examined which relationships remained significant after controlling the influence of other variables through partial correlation. The partial correlation between POM cover and biomass was notably non-significant, and biomass only exhibited a significant but moderate negative partial correlation with water depth (Table 3). POM cover continued to display an appreciable negative partial correlation with both water depth and distance from hill crest, and a lesser positive relationship with seabed slope angle.

## Discussion

We detected substantial spatial variation in both POM cover and megafauna biomass in relation to abyssal hill terrain on the PAP. The results suggested that water depth, terrain type, and distance from hill crest were important in determining the distribution of POM cover at the seabed. The interaction of bottom currents with seabed terrain is most likely the mechanism through which POM cover was controlled. Various measures of local terrain elevation (water depth, BPI, seabed slope angle, etc.) provide reasonable proxy variables for the complex interactions between bottom currents and seafloor features^9^,^19^,^23^,^24^.

The influence of bottom current speed on POM cover may be non-linear. For example, above a threshold speed, POM is resuspended and potentially removed, while below threshold, POM accumulation at the seabed may be positively correlated with current speed. Seafloor time-lapse photography and near-bottom current meter data from the Porcupine Seabight^23^ (4,025 m water depth) showed resuspension of POM at c. 7 cm s^−1^. During the period of our study, current speed was monitored at 30-minute intervals 100 m above the abyssal plain c. 6 km to the east of our seabed survey area^21^ (Supplementary Fig. 4), the mean speed recorded was 3.8 cm s^−1^, with 8% of observations exceeding 7 cm s^−1^, time-integrated flow was to the SE and highest mean current speeds directed to the SSE. Clearly, speeds may have exceeded this threshold more frequently over the elevated terrain features studied. Similarly, current speeds are likely to have been generally reduced in the BBL over the abyssal plain by comparison to the water column 100 m above the plain. Modified bottom water current speed, direction, and turbulence, have been observed over elevated areas elsewhere, including small abyssal hills^5^,^25^,^26^. Our observation of significantly enhanced BBL turbidity over elevated terrain is also suggestive of increased speed/turbulence, and consequently resuspension of POM. Systematic tidal variation in current speed and direction (Supplementary Fig. 5) could introduce a temporal bias to our observations; however, this seems unlikely given the extended period (9-days) of our observations. For example, in our comparisons of the high-resolution study areas (Fig. 1A, Areas A–C), each represents 6-hours of continuous observation, such that the full range of tidal current speed is likely to have been encompassed in each area.

Downslope, gravity-related transport of POM at the seafloor is important on the continental slope, and in major geomorphological features such as canyons and trenches^6^,^7^,^27^. It is conceivable that some of the differences and trends in POM cover we observed were influenced by downslope transport processes. Nevertheless, POM cover was consistently higher on elevated terrain than on the plain in all of our comparisons, suggesting that downslope transport was of limited significance in our case.

Our finding that Hill terrain had 2.5 times the megafauna biomass of Plain terrain was consistent with previous work at the PAP, where a series of three hill summits and one hillside location were found to have a mean megafauna biomass of 3.1 times that of the mean of four plain locations spread up to ~40 km apart^18^. Fishes notably exhibited no observed distribution structure over the hills examined in the present study, as determined from the same AUV missions^20^. However, over 90% of the fish recorded were presumed scavengers including carrion feeders (e.g. Macrouridae), and consequently unlikely to respond to POM cover^20^, i.e. the carrion on which they feed has a lower potential for near-seabed current influence/redistribution. Our megafauna biomass data exhibited substantial negative correlations with water depth and distance from hill crest, though partial correlations suggested that seabed elevation was the strongest single correlate. The observed magnitude of the effect (factor of 2.5, Hill relative to Plain) greatly exceeded that expected from depth-only attenuation of POM at the study site (factor 1.01)^14^, the observed seabed POM cover (factor 1.05), and that expected for megafauna biomass (factor 1.07; Supplementary Text)^2^.

Interpreting these differences in apparent effect scale, 1.01–1.05 in food supply versus 2.5 in standing stock biomass, requires a number of assumptions. By reference to the Metabolic Theory of Ecology^28^, its potential application to seabed biological communities generally^29^, and deep-sea fauna specifically^30^, we can suggest five important points: (a) the megafauna (as quantified here) are likely to represent a substantial fraction of total seafloor biomass; (b) megafauna biomass is likely to scale proportionately with total seafloor biomass; (c) that the PAP megafauna taxa are likely to have lifespans in excess of a decade (typical individual body mass 5 g, environmental temperature 2.5 °C), and consequently; that (d) megafauna biomass is likely to scale directly with integrated multi-year food supply (e.g. POM, phytodetritus); and that (e) biomass links to resource consumption (e.g. respiration)^3^. The apparent scale difference between POM cover (1.05), as a proxy for potential food supply, and megafauna biomass (2.5) enhancement factors from Plain to Hill, therefore suggests that a substantial additional organic matter supply supports the observed Hill biomass–that additional supply is most likely laterally transported POM from the adjacent water column, driven by bathymetrically enhanced bottom current speeds.

We assume that instantaneously observed seabed POM cover represents a dynamic balance between supply (vertical and lateral) rate and removal (decomposition and consumption) rate. For example, the apparent consumption of POM (phytodetritus) by some megafauna taxa can lead to the removal of most visible POM within weeks^31^,^32^. Similarly, resuspended POM may be further consumed within the water column of the BBL, a processes that has previously been observed to occur for 2–3 months following the peak flux period over the PAP^33^. The mechanisms that underpin such bathymetrically-linked relationships are challenging to resolve, in part, because visible accumulations of settling POM on the seafloor are ephemeral often only lasting several weeks, and even then potentially subject to tidal variation^31^,^33^,^34^,^35^. Given that the vertical flux rate is only expected to be a factor of 1.01 higher on the Hill relative to the Plain, and that removal rate may scale directly with megafauna biomass, then a near-corresponding factor increase is implied in laterally transported organic matter supply (i.e. factor 2.4). The potential significance of laterally transported material is supported by our observation of a c. five-fold increase in megafauna suspension feeder biomass from Plain to Hill. Our observation of increased suspended particulate load (turbidity) in the BBL over the hill is consistent with this difference in suspension feeder biomass. Similar, potential links between bathymetrically enhanced current speeds and seafloor biomass have been recorded in other deep-sea environments^5^,^18^,^36^,^37^,^38^.

The likely significance of bathymetrically enhanced bottom current speeds is also suggested in our observations of significant positive correlations between mud, total nitrogen, and total organic carbon content of seabed sediments and water depth–i.e. reduced on Hill relative to Plain, in opposition to the POM and biomass trends. Our results are consistent with previous studies of other hill and plain locations at the PAP^18^,^19^. The reduced sediment mud content on hills reflects winnowing of the sediments by enhanced current speeds (i.e. loss of fine, silt and clay, particles). This effect is very readily observed on PAP abyssal hills by the ubiquitous presence of ice-rafted dropstones at the sediment surface, that are absent from plain sites^18^; post-glacial sedimentation has buried the abyssal plain dropstones, but not those on hills where the sediment column has been winnowed by topographically enhanced currents.

The change in sediment mud content (elevated terrain median 74%, plain median 85%; 0–1 cm sediment horizon) that we have observed is likely accompanied by a change in the dominant sediment mineralogy, as has been previously documented for other hill and plain sites at the PAP^39^. This shift in sediment type and mineralogy may be significant when considering the sediment inventory of organic carbon. TOC is frequently associated with clay particles or minerals^40^, and organic carbon-clay systems are thought to be important in the preservation of organic matter in marine sediments^41^. Our data do exhibit a statistically significant positive relationship between sediment mud content and TOC content (0–1 cm sediment horizon; Spearman’s rank correlation r_s_ = 0.583, n = 21, p = 0.006). Turnewitsch *et al*.^19^ have previously considered organic matter supply to an adjacent much larger hill on the PAP, noting that reduced sedimentary organic carbon content may be the result of both (a) reduced deposition of phytodetritus, and (b) reduced organic matter preservation, both processed being driven by topographically enhanced bottom water current speeds. Our data are certainly consistent with the latter process (b). However, over the modest terrain elevations we have studied in detail, we have not detected any reduction in the deposition of phytodetritus (as assessed by instantaneous seabed POM cover).

Despite limiting our study to very modest terrain elevations (<80 m) in deep water (4850 m), we have detected substantive change in seafloor food supply and megafauna biomass, that appears to be driven by bathymetrically enhanced bottom current speeds^42^. The abyssal hill terrain studied supported a biomass 2.5 times greater than the surrounding abyssal plain, suggesting a corresponding enhancement of biological rate processes and ecosystem functions. Given the prevalence of abyssal hill terrain on our planet, and that water depth-only null predictions yield enhancement factors of only 1.0–1.1, we believe that our observations are particularly significant. We anticipate that these results will be mirrored in other hill areas. We expect that the relative magnitude of the increase in both food supply and biomass may increase, to some extent, with greater seabed elevations, acting through locally enhanced bottom water current speeds.

This new perspective suggests that elevated terrain may experience magnified responses to change in vertical POC flux from surface waters, as may result from climate change^43^. There may also be practical implications for establishing appropriate environmental baselines, and in making impact assessments, over varied bathymetry in abyssal regions subject to seafloor mining^44^. Our results would also suggest the need for caution in global meta-analyses of the relationships between POM cover, or sedimentary TOC, and benthic standing stocks where local seafloor terrain is not considered. We also note that this new appreciation of hills driving seafloor processes does not alter previous interpretations of substantial temporal change in the seafloor community on the plain at PAP^45^.

## Conclusions

Our results demonstrate that changes in bathymetry as small as a few tens of metres can substantially impact the local supply of organic matter to the seafloor and consequently megafauna biomass. There appears to be strong evidence to suggest that this effect is driven by bathymetrically enhanced bottom water currents. The enhanced megafauna biomass on hills is likely mirrored by increased ecological function and carbon cycle roles (e.g. carbon standing stock and total seafloor respiration). Abyssal hill terrain may represent the most widespread landform on the planet^46^, with an estimated 25 million hills of 100–1000 m elevation present globally, and even higher numbers of smaller features suspected^10^. The influence of abyssal hill terrain on deep-sea ecology and carbon cycling is likely to be ubiquitous. Understanding this landscape-scale heterogeneity appears to be vital to the biogeography, ecology and biogeochemistry of the world’s seafloor.

## Methods

During RRS *Discovery* cruise 377, 5–27 Jul 2012, a series of Autonomous Underwater Vehicle (AUV) seabed photographic surveys were carried out covering two spatial scales^47^. One comprised a grid having 1 km line spacing over and around an abyssal hill, with a single line extending a further 10 km across the abyssal plain to the south of the hill. The second scale comprised of three grids with 100 m line spacing, one on the abyssal plain immediately to the north of the abyssal hill, one on the hill, and one on the plain 10 km to the south of the hill (Fig. 1A).

Colour images of the seabed were obtained using a vertically-mounted Point Gray Research Inc. Grasshopper 2 camera, with a 2/3″ sensor (2448 × 2048 pixels), attached to the AUV *Autosub6000*. The camera was fitted with a 12 mm lens, yielding in water acceptance angles of 26.7° and 22.6°. Images were taken every 0.9 seconds from a target altitude of 3.2 m. Full details of the field methodology are given elsewhere^22^.

To estimate megafauna biomass^48^, 64,690 images were mosaicked in groups of 10 to produce 6,469 image tiles each representing ~14 m^2^ of seafloor, these tiles were then randomly assigned to multiple investigators^49^, who annotated the tiles to generate taxon-specific numerical density and body size data (methods detailed elsewhere)^22^. Fresh wet weight megafauna biomass was estimated via morphotype-specific equations relating image-measured body dimension to individual body mass^18^,^50^. Each tile was assigned a terrain category and corresponding environmental variables based on geolocation. Biomass data were log transformed prior to parametric statistical analyses to account for right skew in the data^51^. Total megafauna biomass was partitioned among nominal feeding types based on previous studies of stable isotopes^52^ and individual behaviour^53^ carried out in the near vicinity.

Seabed cover by particulate organic matter (POM) was estimated from individual (un-mosaicked) images. The images were cropped to partially correct for non-uniform illumination, the remaining image (2248 × 1548 pixels) representing ~1.6 m^2^ of seafloor. Percentage seabed cover by POM was estimated using a custom MATLAB (The MathWorks Inc.) routine based on colour image segmentation and resultant object detection measurement (Supplementary Fig. 2; Supplementary Text). Two classes of POM were identified (‘light’ and ‘dark’), this simplified and improved image segmentation, and these two classes were summed to produce ‘total’ POM cover (%). POM data were logit transformed prior to parametric statistical analyses to account for the proportional nature of the data^54^.

Bathymetric data from the survey area were collected during RRS *James Cook* cruise 071, 29 Apr–12 May 2012, using a hull-mounted Kongsberg EM120 12 kHz multibeam system^55^, these data were processed and gridded using Caris HIPS and SIPS software (Teledyne CARIS, Inc.) to give a final bathymetric model resolution of 100 m. The bathymetric model data were further processed to yield seabed slope angle, profile curvature, and rugosity via native functions in ArcGIS v10.2 (Environmental Systems Research Institute). Bathymetric position index (BPI), a second order derivative of the model surface, relating local elevation to the wider landscape, was calculated using the package ‘Benthic terrain modeller’^56^. BPI was calculated in an annulus neighbourhood at a scale factor of 1200 m (i.e. 12 cell outer radius) with an inner radius of four cells (400 m), and combined with seabed slope angle to distinguish three primary terrain classes: ‘Hill’, ‘Slope’, and ‘Plain’ (Table 1, Fig. 1B). Finer divisions of BPI and slope angle were employed to produce a secondary classification comprising twelve terrain types (Supplementary Fig. 1).

Variations in megafauna biomass and POM seabed cover were assessed with water depth and terrain variables. Images and tiles were binned into natural 12.5 m depth intervals (i.e. integer multiples of 12.5); to avoid low sample size in the first and last bins, data were amalgamated with adjacent bins (Table 1). Each image and tile was assigned a primary and secondary terrain class based on geolocation. After transformation (see above), both POM cover and megafauna biomass exhibited significant inequalities in variance between environmental categories (p < 0.001; Levene’s test^57^). To acknowledge this heteroscedasticity, and the unbalanced samples sizes, we employed Welch’s modification of the one-way ANOVA^58^, and used the Games-Howell method for subsequent pair-wise comparisons^59^. We also considered effect size using Cohen’s *d*-statistic^60^. The potential impact of spatial autocorrelation on our analyses and interpretations was addressed by examination of variograms that suggested the phenomenon occurred but was unlikely to impact our interpretations (Supplementary Text; Supplementary Fig. 3). We compared our field estimates of biomass and POM cover with water depth only-driven null predictions (Supplementary Text).

For assessment with terrain variables and classes, seabed POM cover and megafauna biomass were averaged in spatial grid cells corresponding with those of our bathymetric model. Only cells with ≥50 images (POM) and ≥5 tiles (biomass) were included in these analyses. Potential correlations between POM, biomass, and individual terrain parameters were assessed via Spearman’s rank correlation^61^. Partial Spearman’s rank correlation coefficients were also examined (implemented in R^62^ using package ‘ppcor’, function ‘pcor’^63^) to provide additional insight into the practical significance of the observed correlations.

The *Autosub6000* vehicle was fitted with a Seapoint Turbidity Meter (Seapoint Sensors, Inc.), detecting light (880 nm) scattered (15–150° scattering angle) by suspended particles in close proximity to the sensor (<5 cm). Sensor data, calibrated to Formazin Turbidity Units (FTU), were recorded at 2-second intervals throughout the course of our surveys. For assessment these data were binned into natural 12.5 m depth intervals (as for analysis of POM cover and megafauna biomass) and assigned to two groups: (a) water column, where vehicle altitude above seafloor was >10 m, and (b) benthic boundary layer (BBL), where vehicle altitude was ≤10 m.

Seabed sediments were sampled at 21 locations (Fig. 1A; Supplementary Table 2) using a Bowers & Connelly Megacorer^64^ during RRS *Discovery* cruise 377^47^. Sediment particle size distributions were determined by laser diffraction (Malvern Mastersizer)^18^ and results reported as mud content (% wt/wt, particles <63 μm) for the 0–10 mm sediment depth horizon. Sediment total nitrogen (TN) and total organic carbon (TOC) content (% wt/wt) were analysed in duplicate for the 0–10 mm sediment depth horizon using the vapour-phase acid de-carbonation method^65^. These mud, TN and TOC data were assessed for interrelationships and relationships with water depth via Spearman’s rank correlation^61^, and potential relationships illustrated as simple least squares linear fits (Fig. 2D–F). Comparisons between abyssal plain (sites >4840 m) and elevated terrain (sites <4840 m) locations were carried out using Mood’s median test^61^, with confidence intervals calculated using the Wilcoxon signed rank method^66^, both as implemented in Minitab 17 (Minitab, Inc.).

## Additional Information

**How to cite this article**: Morris, K. J. *et al*. Landscape-scale spatial heterogeneity in phytodetrital cover and megafauna biomass in the abyss links to modest topographic variation. *Sci. Rep.* **6**, 34080; doi: 10.1038/srep34080 (2016).

## Supplementary Material

Supplementary Information

## Figures and Tables

**Table 1:** Mean particulate organic matter (POM) cover and megafauna biomass in water depth and terrain classes.

	n	Mean total	n	Mean biomass		
		POM (%)		(g fwwt m^−2^)		
		(95% CI)		(95% CI)		
Depth band (m)						
<4788	1035	45.5 (45.2–45.8)	256	5.5 (4.9–6.2)		
4788–4800	2381	45.8 (45.6–45.9)	400	5.5 (4.9–6.1)		
4800–4813	3092	45.2 (45.1–45.4)	352	5.1 (4.6–5.6)		
4813–4825	2920	45.6 (45.5–45.8)	347	5.7 (5.0–6.4)		
4825–4838	3360	45.2 (45.0–45.3)	459	3.6 (3.1–4.1)		
>4838	79560	43.4 (43.4–43.4)	4654	2.1 (2.0–2.2)		
1° Terrain class						
Hill	6912	45.4 (45.2–45.6)	1207	5.2 (5.0–5.6)		
Slope	9437	44.7 (44.6–44.7)	1016	3.4 (3.1–3.7)		
Plain	75999	43.4 (43.4–43.4)	4245	2.1 (2.0–2.2)		
2° Terrain class					**BPI**	**Slope angle**
Hill B	147	45.1 (44.3–45.9)	43	4.1 (2.8–5.9)	LT -100	2° to 5°
Hill D	3457	44.9 (44.8–45.0)	424	4.9 (4.4–5.4)	−100 to −25	LT 2°
Hill E	3308	45.5 (45.4–45.7)	731	5.5 (5.1–5.9)	−100 to −25	2° to 5°
Slope A	6287	44.5 (44.4–44.6)	625	3.4 (3.0–3.8)	−25 to 50	2° to 5°
Slope C	2932	44.9 (44.7–45.0)	320	3.4 (2.9–4.0)	GT 50	LT 2°
Slope D	218	45.2 (44.7–45.8)	71	3.4 (2.4–4.7)	GT 50	2° to 5°
Plain	75999	43.4 (43.4–43.4)	4245	2.1 (2.0–2.2)	−25 to 50	LT 2°

Bathymetric position index (BPI) and seabed slope angle define terrain classes as indicated (LT, less than; GT greater than; full classification is provided with Supplementary Fig. 1). (n, number of POM images or number of biomass tiles; fwwt, fresh wet weight; 95% CI, confidence interval of mean value).

**Table 2:** Sediment total organic carbon (TOC), total nitrogen (TN), carbon-to-nitrogen ratio (C/N), and mud (particles <63 μm) content.

	Sites	n	Median	Confidence		Correlation with depth		Comparison by elevation	
				(interval)	level	r_s_	p	χ^2^	p
TOC (%)	All	21	0.336	(0.317–0.354)	94.8%	0.680	<0.002	5.74	<0.020
	>4840 m	9	0.361	(0.338–0.386)	95.6%				
	<4840 m	12	0.313	(0.294–0.336)	94.5%				
TN (%)	All	21	0.059	(0.056–0.063)	94.8%	0.706	<0.001	10.75	<0.002
	>4840 m	9	0.065	(0.061–0.067)	95.6%				
	<4840 m	12	0.055	(0.051–0.059)	94.5%				
C/N	All	21	5.734	(5.471–5.925)	94.8%	−0.162	ns	1.29	ns
	>4840 m	9	5.698	(5.378–6.046)	95.6%				
	<4840 m	12	5.788	(5.396–6.116)	94.5%				
Mud (%)	All	21	79.8	(73.2–85.8)	94.8%	0.476	<0.050	5.74	<0.020
	>4840 m	9	85.3	(79.5–91.5)	95.6%				
	<4840 m	12	74.0	(65.7–84.2)	94.5%				

Median and c. 95% confidence intervals (level, achieved confidence level) for all samples, abyssal plain samples (>4840 m), and elevated terrain samples (<4840 m). Also indicated are: Spearman’s rank correlation (r_s_, and associated p value) of parameter with water depth, and (ii) Mood’s median test (χ^2^, and associated p value) comparison of abyssal plain with elevated terrain sample values.

**Table 3:** Simple and partial Spearman’s rank correlations of particulate organic matter (POM) cover and megafauna biomass with selected seabed terrain-related variables.

	All grid cells		Common grid cells (n = 309)			
	Simple correlation		Simple correlation		Partial correlation	
	POM(n = 779)	Biomass(n = 471)	POM	Biomass	POM	Biomass
POM	—	—	—	0.161*	—	−0.013
Biomass	—	—	0.161*	—	−0.013	—
Water depth	−0.537***	−0.602***	−0.535***	−0.219***	−0.412***	−0.150*
Distance	−0.623***	−0.441***	−0.631***	−0.163**	−0.465***	−0.081
Slope	0.405***	0.383***	0.564***	0.152*	0.177**	0.019
Rugosity	0.257***	0.150***	0.379***	0.092	−0.078	−0.014
Curvature	−0.012	0.031	−0.050	−0.002	−0.042	0.003

(Distance, distance from hill crest; n, number of map grid cells [100 × 100 m] contributing data; statistical significance, *p < 0.01, **p < 0.005, ***p < 0.001).
