# Supplementary material for: Landscape-scale spatial heterogeneity in phytodetrital cover and megafauna biomass in the abyss links to modest topographic variation
Source: Sci Rep. 2016 Sep 29;6:34080. doi: 10.1038/srep34080 (PMC5040962; doi:10.1038/srep34080)
Supplement: Supplementary Information [file srep34080-s1.pdf]

Supplementary Information for:

Landscape-scale spatial heterogeneity in phytodetrital cover and megafauna biomass in the abyss links to modest topographic variation

Kirsty J Morris[1*], Brian J Bett[1], Jennifer M Durden[1,2],

Noelie MA Benoist[1,2], Veerle AI Huvenne[1], Daniel OB Jones[1],

Katleen Robert[1,2], Matteo C Ichino[1,2], George A Wolff[3] and Henry A Ruhl[1]

1 National Oceanography Centre, University of Southampton Waterfront Campus, European Way, Southampton SO14 3ZH, UK.

2 Ocean and Earth Science, University of Southampton, National Oceanography Centre Southampton, European Way, Southampton SO14 3ZH, UK.

3 School of Environmental Sciences, University of Liverpool L69 3BX, UK.

*Corresponding Author email: K.Morris@noc.ac.uk

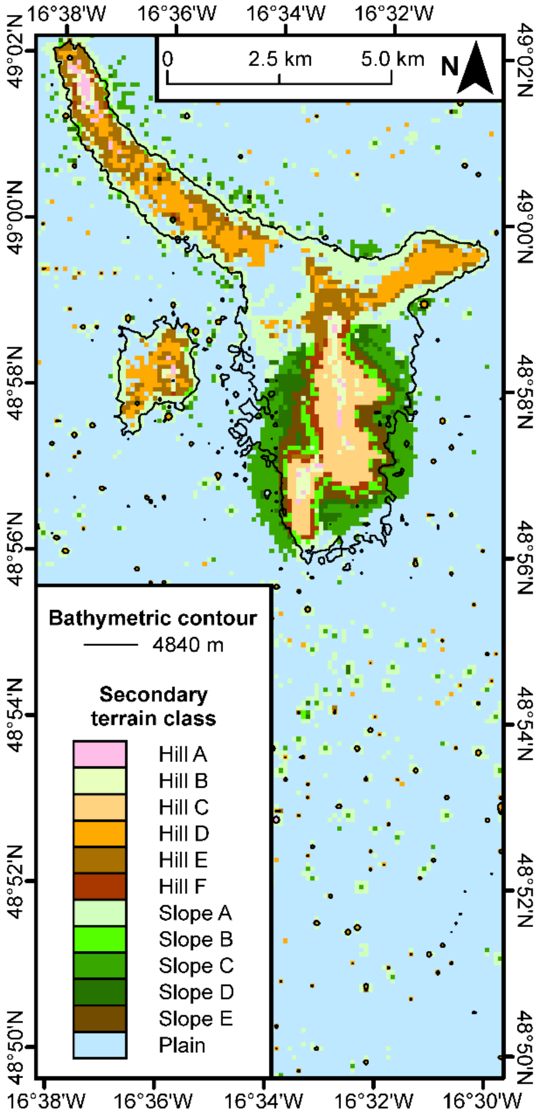

Supplementary Figure 1. Porcupine Abyssal Plain study area. The defined secondary terrain types and the primary hill-bounding bathymetric contour (4840 m) are illustrated. Map projection is UTM WGS 1984 zone 28N (ArcGIS v10.2, Environmental Systems Research Institute). The classification criteria were as follows:

Bathymetric position index (BPI)	Seabed slope angle		
	<2°	2° to 5°	>5°
**Less than -100**	Hill A	Hill B	Hill C
**-100 to -25**	Hill D	Hill E	Hill F
**-25 to 50**	Plain	Slope A	Slope B
**Greater than 50**	Slope C	Slope D	Slope E

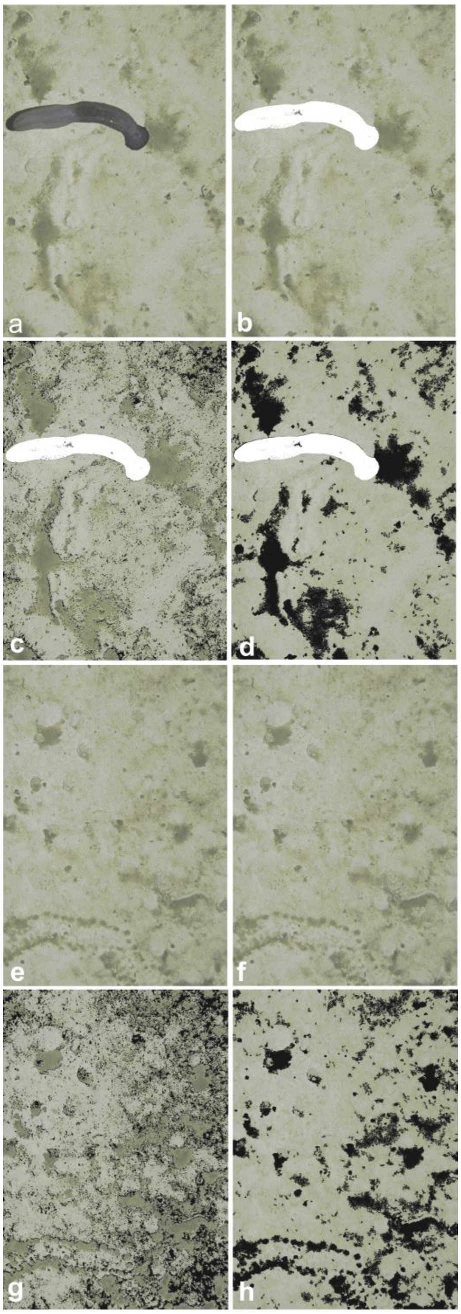

Supplementary Figure 2. Examples from the processing of two images to illustrate the particulate organic matter (POM) detection routine. Image 1: (a) Original image. (b) Removal of dark area (specimen of sea cucumber, *Psychropotes* sp.). (c) Detection of 'light' POM cover. (d) Detection of 'dark' POM cover. Image 2: (e) Original image. (f) Dark area removal (no effect in this case). (g) 'Light' POM detection. (h) 'Dark' POM detection.

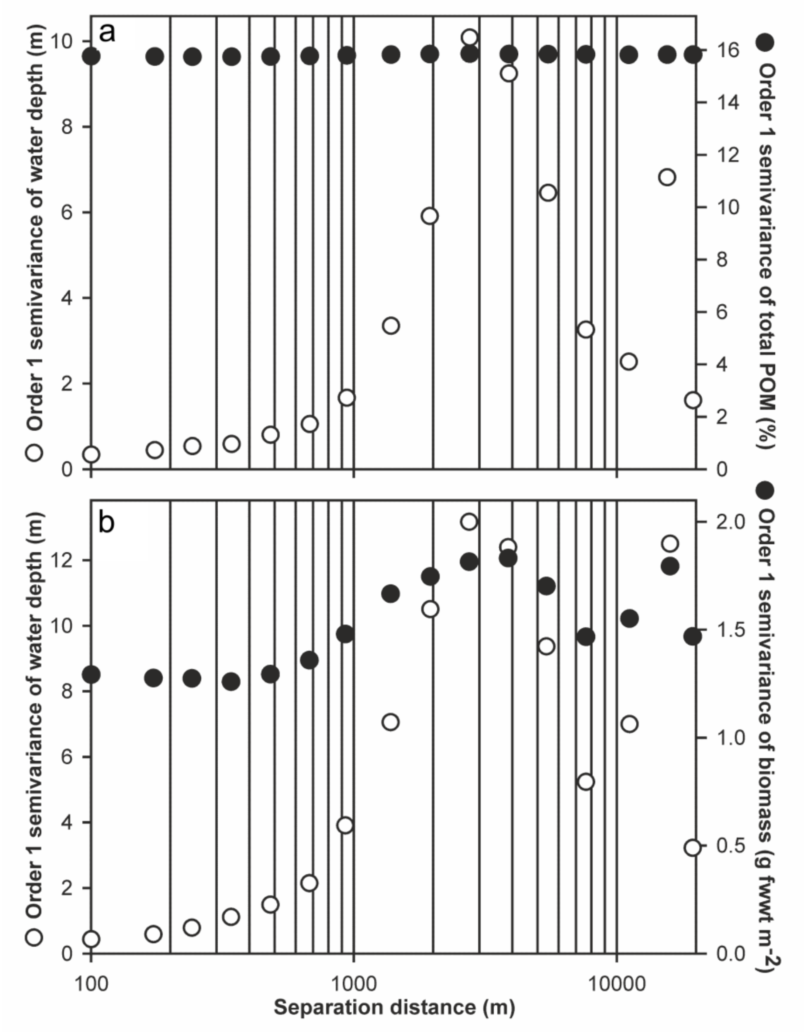

Supplementary Figure 3. Madograms (order 1 semivariance) of (a) total POM cover and (b) megafauna biomass. In both cases shown with corresponding variograms of water depth (○) for comparative reference.

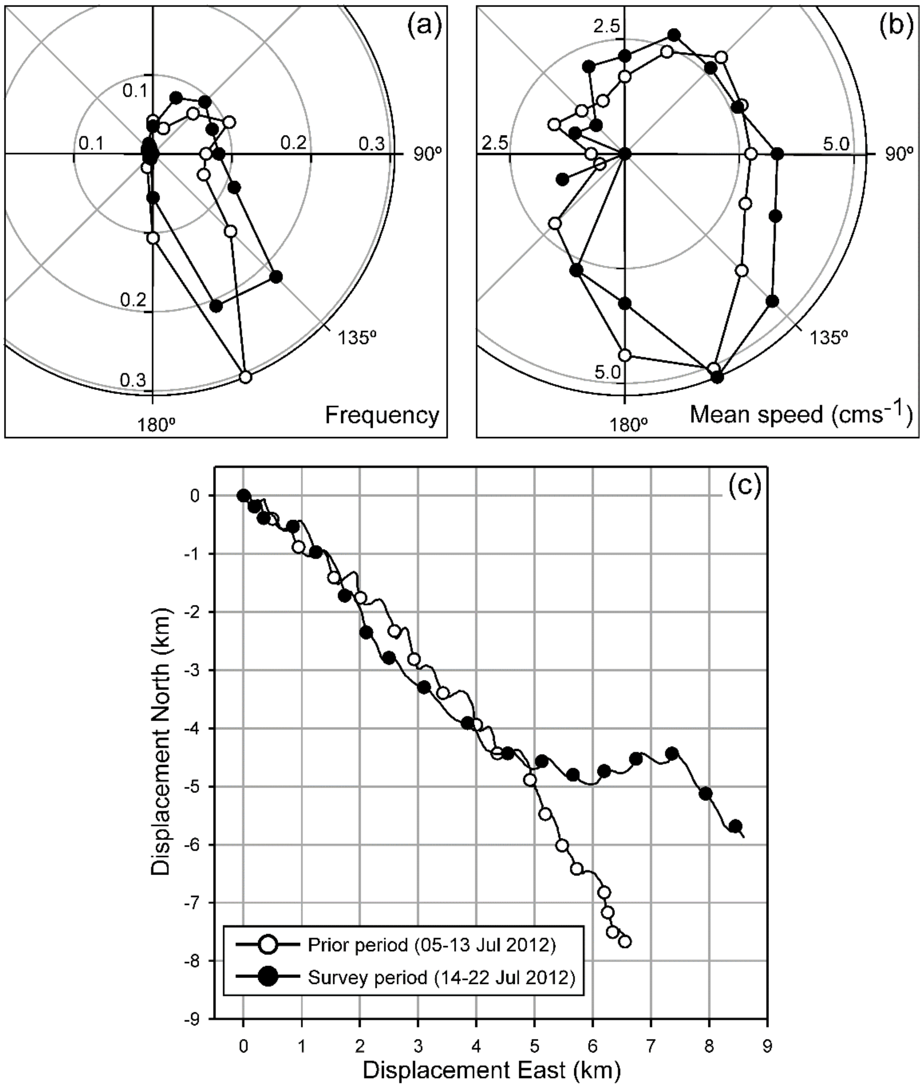

Supplementary Figure 4. Graphical summaries of bottom water current speed and direction during, and prior to, seabed survey. (a) Polar plot of frequency of current direction. (b) Polar plot of mean current speed. (c) Progressive vector plot, symbols represent 12.5-hour periods. In all cases the data refer to current speed and direction recorded 100 m above the abyssal plain (sounding 4850 m) in the 9-day period prior to, and the 9-day period of the seabed survey. The current meter data were recorded at a position some 6 km to the east of the centre of the seabed survey area on a sediment trap mooring (NOC station number JC071-042) operated as part of the Porcupine Abyssal Plain Sustained Observatory programme[4].

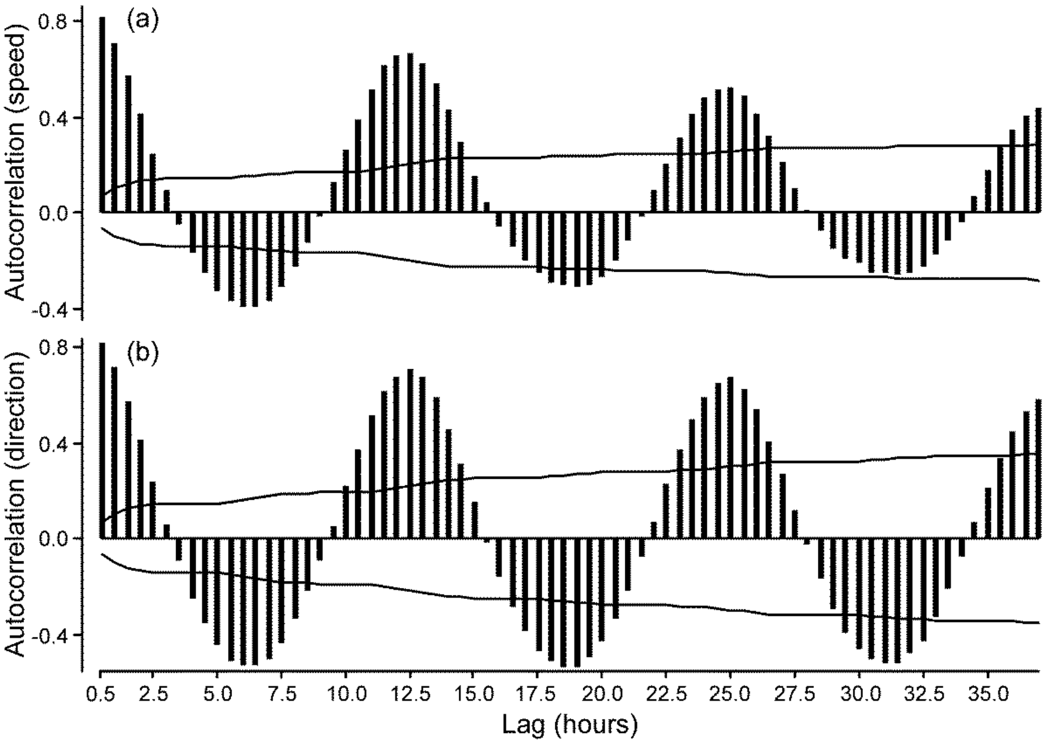

Supplementary Figure 5. Tidal component of bottom water current speed and direction during the period 05-22 July 2012. (a) Autocorrelation of current speed (bars). (b) Autocorrelation of current direction (bars). Line plots indicate 95% confidence interval of zero correlation [i.e. bars extending beyond lines indicate significant autocorrelation]. Data are as described in Supplementary Fig. 4.

	***F* (v1,v2)**	***p***	**R^2 (%)**	**Games-Howell pairwise comparison groups**						
**Full survey depths**				<4778	4788 -4800	4800 -4813	4813 -4825	4825 -4838	>4838	
POM	556 (5, 5151)	<0.001	3.41	A, B	A	B	A	B	C	
Biomass	145 (5, 956)	<0.001	7.63	A	A	A	A	B	C	
**Area D depths**				<4778	4788 -4800	4800 -4813	4813 -4825	4825 -4838	>4838	
POM	53 (3, 529)	<0.001	1.31				A	B	C	
Biomass	16 (3, 13)	<0.001	4.97				A	B	B	
**High resolution areas**				N Plain (Area-A)	Hill (Area-B)	S Plain (Area-C)				
POM	1427 (2,10218)	<0.001	8.74	A	B	C				
Biomass	159 (2, 646)	<0.001	7.42	A	B	A				
**Primary terrain class**				‘Hill’	‘Slope’	‘Plain’				
POM	969 (2, 1178)	<0.001	2.37	A	B	C				
Biomass	323 (2, 2272)	<0.001	6.70	A	B	C				
**Secondary terrain class**				Hill-B	Hill-D	Hill-E	Slope-A	Slope-C	Slope-D	Plain
POM	308 (6, 1271)	<0.001	2.28	A, B, C	B	A	C	B	B	D
Biomass	96 (7, 125)	<0.001	6.72	A, B	A	A	B	B	A, B, C	C

Supplementary Table 1. Statistical comparisons of total seabed particulate organic matter (POM) cover, and megafauna biomass across depths and areas. (*F*, from Welch’s one-way ANOVA; v1, v2, corresponding degrees of freedom; *p*, probability; R^2, adjusted coefficient of determination; A-D, non-significant ($p > 0.05$) pairwise comparison groups, i.e. comparisons that do not share a common letter are significantly different ($p < 0.05$)).

Sample station number	Latitude (°N)	Longitude (°E)	Water depth (m)	Mud content (0-10 mm, %)	Total nitrogen content (0-10 mm, %)	Total organic carbon content (0-10 mm, %)
D377-010	49.0189	-16.5532	4844	95.7	0.067	0.384
D377-011	48.9925	-16.5271	4820	75.8	0.066	0.349
D377-014	48.9820	-16.5468	4779	74.0	0.059	0.343
D377-017	49.0198	-16.6063	4847	83.3	0.066	0.375
D377-019	48.9383	-16.6032	4844	86.8	0.062	0.336
D377-020	48.9369	-16.6125	4844	85.0	0.068	0.345
D377-021	48.9503	-16.5850	4845	84.8	0.063	0.359
D377-022	48.9520	-16.5344	4845	72.4	0.062	0.318
D377-023	48.9765	-16.5477	4773	80.0	0.059	0.296
D377-024	48.9635	-16.5530	4715	70.3	0.050	0.294
D377-025	48.9510	-16.5501	4718	61.4	0.051	0.328
D377-027	48.9665	-16.5454	4695	71.3	0.048	0.288
D377-029	48.9560	-16.5454	4633	57.6	0.050	0.322
D377-030	48.9631	-16.5476	4668	87.3	0.052	0.300
D377-031	48.9540	-16.5454	4674	49.5	0.057	0.280
D377-036	48.9601	-16.5451	4693	73.8	0.055	0.288
D377-037	48.9632	-16.5589	4787	98.3	0.065	0.377
D377-038	48.8385	-16.5184	4844	98.1	0.056	0.339
D377-040	48.9980	-16.5858	4828	94.4	0.051	0.323
D377-041	48.8352	-16.5150	4844	86.5	0.066	0.422
D377-048	48.8326	-16.5203	4844	83.8	0.066	0.386

Supplementary Table 2. Geolocation and water depth of seabed sediment sampling sites, with determination of mud content, total nitrogen (mean of duplicates), and total organic carbon (mean of duplicates).

Supplementary Text

Predicting megafauna biomass based on water depth alone. We employed the global dataset compiled by Wei et al.[1] to provide a null prediction of the change in megafauna biomass expected by change in water depth (presumed change in vertical POC flux rate) alone. Wei et al.'s prediction:

$$'Megafauna'\ biomass\ \approx 10^{-0.307*Z}$$

(where Z is water depth in kilometres) includes data on fish biomass, and a large proportion of data from shallow-water continental shelf and continental margin studies. Consequently, we have taken the data provided as Appendix 1 of Wei et al.[1] and selected megafauna invertebrate-only biomass data, and limited the included studies to those from water depths ≥1000 m, in an attempt to best match our own megafauna invertebrate-only data from an open ocean location. The remaining biomass data (n = 130) were $\log_{10}$ transformed and regressed on water depth (km) to yield our derived prediction,

$$Megafauna\ biomass\ \approx 10^{-0.448*Z}$$

The resultant regression was significant ($F[1,128] = 56.58$, $p < 0.001$, $R^2_{\mathrm{adjusted}} = 30.7\%$), the depth coefficient having a 95% confidence interval (CI) of -0.330 to -0.566. We used these results to estimate our null prediction of the depth-only variation in megafauna biomass, e.g. from our deepest (abyssal plain) to shallowest (hill) depth band a predicted increase factor of 1.069 (95% CI 1.050-1.087).

Particulate organic matter (POM) detection routine. Fifty images were randomly selected, opened in MATLAB, visualised in the "Color Thresholder App" in HSV (hue, saturation, value) colour space, and S and V levels manually tuned to identify areas of 'dark' POM. The same process was repeated for 'light' POM. Light and dark POM were analysed separately to improve discrimination from the background (POM absent) seafloor. Manually tuned S and V values were averaged across the 50 trial images and these mean levels used in the automated process. Early testing indicated that false positive POM detection resulted from the presence of dark shadows or megafauna specimens (e.g. *Psychropotes* sp.). Consequently, an additional filter was applied to identify and remove these "dark" areas prior to the POM quantification process (see example in Supplementary Fig. 2).

Analysis was limited to images in the 3.0 - 3.3 m altitude range to improve consistency in seabed illumination, providing 92,348 images for analysis. Detected POM areas were further processed with the MATLAB functions 'bwconncomp' and 'regionprops' to identify and

remove regions of less than 70 contiguous pixels, to eliminate potentially spurious detections. The sum of light and dark POM cover data was found to have a small but systematic bias with AUV altitude. Consequently, this bias was removed be re-centring the data in fine altitude bins (10 cm bin sizes).

Spatial autocorrelation. Following Tobler's first law of geography ("everything is related to everything else, but near things are more related than distant things")[2], it was important for us to consider the potential impact of spatial autocorrelation on our analyses and interpretations of spatial variation in particulate organic matter (POM) cover and megafauna biomass. We examined this issue by constructing sampled, empirical madograms[3] of these variables, and of water depth as a key reference variable in our study. Madograms represent variation in the order 1 semivariance of the variable of interest with distance of separation between pairs of observations. Order 1 semivariance was calculated as half the average absolute difference between pairs of sample points, in our case we examined 500,000 pairs of points selected at random without replacement from our much larger datasets. These random point pairs were then grouped into horizontal spatial separation distance bins (√2 geometric classes) and the semivariance estimated for each distance bin.

The resultant madograms are illustrated in Supplementary Fig. 3. The variograms of water depth derived from both datasets (Supplementary Fig. 3 a and b) similarly recorded two peaks in semivariance at separation distances of c. 3 and 16 km, these were consistent with local and broadscale hill-to-plain comparisons within our data, e.g. between high resolution survey areas A and B (3 km), and C and B (16 km). In marked contrast, the variogram for total POM cover was practically invariant across the full spatial scale considered (Supplementary Fig. 3 a). This was not surprising given the near uniform average POM cover across the depth and terrain categories we analysed (see main text). Consequently, spatial autocorrelation in total POM cover is unlikely to have had any practical impact on our interpretations. We should, however, note that very obvious spatial aggregation (i.e. strong positive spatial autocorrelation) of total POM cover was evident at the centimetric-scale, driven by biogenic topography (see e.g. Supplementary Fig. 2).

Inspection of the megafauna biomass madogram (Supplementary Fig. 3 b) did indicate a spatial autocorrelation structure that appeared to be closely matched to that of the water depth variogram, i.e. peaks in semivariance at separation distances of c. 3 and 16 km. Although there was clear spatial autocorrelation in our megafauna biomass data, it appeared to be very closely linked to topographic elevation (i.e. the primary focus of our study), such

that it is unlikely to have had any practical impact on our interpretations. We should finally note that homogeneity of variance was not assumed in any of the parametric analyses undertaken of spatial variation in megafauna biomass or POM cover (see main text Methods).

Supplementary References

1. Wei, C.-L., et al. Global patterns and predictions of seafloor biomass using random forests. *PLoS ONE* **5**, e15323, 10.1371/journal.pone.0015323 (2010).
2. Tobler, W. A. Computer movie simulating urban growth in the Detroit region. *Econ. Geogr.* **46**, 234-240 (1970).
3. Cooley, D., Naveau P. & Poncet, P. Variograms for spatial max-stable random fields. In: *Dependence in Probability and Statistics*. Bertail P., Soulier P., Doukhan P. (eds). New York, NY, Springer New York pp 373-390. (2006)
4. Hartman, S. E. et al. The Porcupine Abyssal Plain fixed-point sustained observatory (PAP-SO), variations and trends from the Northeast Atlantic fixed-point time-series. *I. C. E. S. J. Mar. Sci.* **69**, 776–783 (2012).
